# optimalFlow: optimal transport approach to flow cytometry gating and population matching

**DOI:** 10.1186/s12859-020-03795-w

**Published:** 2020-10-27

**Authors:** Eustasio del Barrio, Hristo Inouzhe, Jean-Michel Loubes, Carlos Matrán, Agustín Mayo-Íscar

**Affiliations:** 1 Departamento de Estadística e Investigación Operativa, Universidad de Valladolid, Calle Paseo de Belén, Valladolid, Spain; 2 IMUVA, Calle Paseo de Belén, Valladolid, Spain; 3 Université Paul Sabatier, Route de Narbonne, Toulouse, France; 4 IMT, Route de Narbonne, Toulouse, France

**Keywords:** Flow cytometry gating, Optimal transport, Wasserstein distance, Clustering, Supervised classification

## Abstract

**Background:**

Data obtained from flow cytometry present pronounced variability due to biological and technical reasons. Biological variability is a well-known phenomenon produced by measurements on different individuals, with different characteristics such as illness, age, sex, etc. The use of different settings for measurement, the variation of the conditions during experiments and the different types of flow cytometers are some of the technical causes of variability. This mixture of sources of variability makes the use of supervised machine learning for identification of cell populations difficult. The present work is conceived as a combination of strategies to facilitate the task of supervised gating.

**Results:**

We propose *optimalFlowTemplates*, based on a *similarity distance* and *Wasserstein barycenters*, which clusters cytometries and produces prototype cytometries for the different groups. We show that supervised learning, restricted to the new groups, performs better than the same techniques applied to the whole collection. We also present *optimalFlowClassification*, which uses a database of gated cytometries and optimalFlowTemplates to assign cell types to a new cytometry. We show that this procedure can outperform state of the art techniques in the proposed datasets. Our code is freely available as *optimalFlow*, a Bioconductor R package at https://bioconductor.org/packages/optimalFlow.

**Conclusions:**

optimalFlowTemplates + optimalFlowClassification addresses the problem of using supervised learning while accounting for biological and technical variability. Our methodology provides a robust automated gating workflow that handles the intrinsic variability of flow cytometry data well. Our main innovation is the methodology itself and the optimal transport techniques that we apply to flow cytometry analysis.

## Background

Flow cytometry (FC) works with ‘high-dimensional quantitative measurement of light scatter and fluorescence emission properties of hundreds of thousands of individual cells in each analysed sample’ (see [1]). These quantitative measurements allow to analyse and classify individual cells, facilitating diverse applications. For example, as mentioned in [2], ‘flow cytometry is used to identify and quantify populations of immune cells’ in order to monitor the immune state of patients or to detect relevant biomarkers by comparing flow cytometries from different patient groups.

A main component in FC is gating, the assignment of individual cells (data records) into discrete cell types. Manual gating, where an expert assigns cell types (labels) to individual cells using a set of rules on one- or two-dimensional projections, has been the prevalent option. However, this manual approach has some shortcomings. Firstly, it is subjective, since it depends on the expertise of the user, on the sequence of markers (measured variables) used to do the projections and on the locations of the gates on those projections. Secondly, it can be very time consuming because it is ‘roughly quadratic in the number of markers’ (see [3]). Lastly, the recent increase in the number of markers and number of cells per cytometry makes human error a relevant factor.

To avoid some of the difficulties related to manual gating there have been different approaches to automated gating. In unsupervised methods there is no need for previously gated cytometries, and gating is done through a clustering procedure. Examples of such methods include CCST [4], which uses a nonparametric mixture model clustering and a data-derived decision tree representation for gating; FLOCK [5], which does grid-based density estimation (with merging) and then applies k-means; FLAME [6], which performs skew *t* model-based clustering; and flowClust [7, 8], which does robust-based clustering through *t* mixture models with Box-Cox transformation. Other related clustering procedures are: flowPeaks [9], which performs Gaussian mixture model-based clustering (with modified covariances) and merging, and flowMeans [10] which does k-means with initialization via mode detection through kernel density-based estimation. More information about state-of-the-art methods can be found in [1, 2].

The accuracy of cell type assignation can be improved using supervised machine learning which takes advantage of the historical information contained in previously gated cytometries (manually or otherwise). Recently, some methods have been produced addressing this problem. DeepCyTOF [3] combines de-noising, deep-learning algorithms, and domain adaptation. flowLearn [11] combines density features of the data, manually selected gating thresholds, and derivative-based density alignments. We stress that other more classical approaches for supervised learning are also available. For example, random forest algorithms, support vector machines or quadratic discriminant analysis can be used when learning from some previously gated cytometry. Supervised machine learning is a well-documented topic and for more detailed explanations we refer to [12].

There are two main setups for using supervised learning in the FC context which are relevant in practical studies. Firstly, the classical one, where there is an available database of historical information. This means that a collection of gated flow cytometries is available and this information can be used to gate a new cytometry. In a second scenario, we have a collection of ungated cytometries, and we want to gate manually (or otherwise) a minimal amount of them and use these gated cytometries to classify the rest. In both setups, there is a fundamental problem intrinsic to FC. That is, flow cytometry data have considerable technical and biological variability, which makes the use of supervised learning difficult. Biological variability is due to intrinsic differences between individuals such as health status, age, gender, etc. Technical variability appears due to different experimental adjustments, variation of conditions during experiments and the use of different measuring devices (flow cytometers).

In this work we provide novel methods for grouping (clustering) gated cytometries. The goal is to produce groups (clusters) of cytometries that have lower variability than the whole collection and, furthermore, that are coherent enough to be considered as a typology by themselves. This, in turn, allows to greatly improve the performance of any supervised learning procedure. We provide evidence of this below. Once we have a partition (clustering) of a collection of cytometries, we provide several methods for obtaining an artificial cytometry (prototype, template) that represents in some optimal way the cytometries in each respective group. These prototypes can be used, among other things, to match populations between different cytometries as suggested in [13, 14] or to analyse and extract characteristics of a group of similar cytometries. In addition, a procedure capable of grouping similar cytometries could help to detect individuals with a common condition, i.e., a sickness, such as cancer. In our work we show that this indeed happens.

*optimalFlowTemplates* is our procedure for clustering cytometries and obtaining templates. It is based on recent developments in the field of optimal transport such as a *similarity distance* between clusterings, introduced in [15], and a *barycenter* (Frechet mean, see [16, 17]) and *k-barycenters* (see [18–20]) of probability distributions.

Additionally, we introduce a supervised classification tool, *optimalFlowClassification*, for the case when a database of gated cytometries is available. The procedure uses the prototypes obtained by optimalFlowTemplates on the database. These are used to initialise *tclust*, a robust extension of k-means that allows for non-spherical shapes, to gate a new cytometry (see [21], not to be confused with TCLUST [22]). By using a similarity distance between the best clustering obtained by *tclust* and the artificial cytometries provided by optimalFlowTemplates we can assign the new cytometry to the most similar template (and the corresponding group of cytometries). We provide several options to assign cell types to the new cytometry using the most relevant information, represented by the assigned template and the respective cluster of cytometries.

Our approach fits into the following general steps:

0. Obtain a database of gated cytometries. This can be done by manual gating or by a suitable automatic gating procedure.
1 Obtain a partition of the database of gated cytometries based on some similarity/dissimilarity measure between gated cytometries.
2 Obtain an artificial prototype (template cytometry) for every cluster of gated cytometries obtained in the previous step.
3 Assign a new ungated target cytometry to the most similar artificial prototype. Use that prototype or the corresponding group of gated cytometries to gate the target cytometry.

Previous approaches fit into this scheme but have implemented it using different methods. The crucial points in all procedures are to define an appropriate similarity/dissimilarity measure and to propose a method for producing templates. We briefly discuss some of the most relevant approaches for our work.

In [23] the Earth Mover’s (Wasserstein, Kantorovich–Rubinstein) Distance was proposed as an appropriate measure of similarity between gated cytometries. This approach is very attractive since Step 1 can be done comparing directly appropriate non-parametric approximations of the distribution of gated (or ungated) cytometries. Additionally, the similarity criterion is a parameter-free distance with a nice interpretation. However, this approach is very computationally intensive and even unfeasible in the high dimension and high cell count setting that is characteristic of modern FC.

QFMatch [24] introduces a similarity between cytometries based on a multidimensional extension of a Quadratic Form (QF)-based distance. This QF-based distance is calculated through a non-parametric approximation of the distributions of the whole gated cytometry and of the separate cell types. Specifically, it is based on the Euclidean distance of the centers of mass between bins of the approximate pooled distribution and on the frequency difference in the bins of the separate cytometries. However, defining a QF-based distance is not trivial and no proposal or suggestion for a method of obtaining templates is made.

The most closely related method to ours is flowMatch [13]. There, similarity between gated cytometries is obtained solving a matching problem known as Generalized Edge Cover (GEC) and templates are obtained merging matched vertices.

Our proposal builds on the best properties of the previous procedures. It offers a similarity measure between gated cytometries (2) which is a distance and is parameter free as QFMatch [24]. The similarity is based on optimal transport which is well suited for FC [23] and has a very intuitive meaning. Through the optimal transport soft assignment problem (1), we have extensive freedom for choosing the cost of transporting cell types from one cytometry to another in a fairly straightforward way, as in flowMatch. This enables us to measure the difference between the distributions of cell types in different cytometries using suitable parametric or non-parametric approximations. We promote the use of the 2-Wasserstein distance and location-scale mixture models to be able to obtain meaningful templates. These are not based on pooling or keeping clusters of cell types, as for example in flowMatch, but in obtaining a prototype that is a consensus between all the cytometries that are in the same group. The template simultaneously represents a consensus for the location, shape, and proportion of the different cell types present in the group of cytometries.

## Methods

We can view a gated flow cytometry, $X^i$, as a collection of $n_i$ multidimensional points with their associated labels (cell types or group labels) forming a set $L^i=\{L_k^i\}_{k=1}^{k_i}$ of $k_i$ different labels. Hence, a gated cytometry can be described as $X^i = \{(X^i_j,Y^i_j)\}_{j = 1}^{n_i}$ where $X^i_j\in {\mathbb {R}}^d$ and $Y^i_j\in L^i$. Alternatively, we could describe it as a partition (clustering) of all $X^i_j$ into groups (clusters) formed by points sharing the same labels. That is, ${\mathcal {C}}^i=\{({\mathcal {C}}^i_k, p^i_k)\}_{k=1}^{k_i}$ where ${\mathcal {C}}^i_k = \{X^i_j:1\le j\le n_i,Y_j^i = L^i_k\}$ is a cluster and $p^i_k$ is a weight associated with label $L_k^i$. A third useful description is to view a gated cytometry as a clustering but coming from a mixture of location-scatter multivariate distributions. With some abuse of notation ${\mathcal {C}}^i = \{(m^i_k,S^i_k,p^i_k)\}_{k=1}^{k_i}$ where $m_k^i,S^i_k$ are the multivariate mean and covariance of the points in cluster ${\mathcal {C}}^i_k$.

**Fig. 1:**
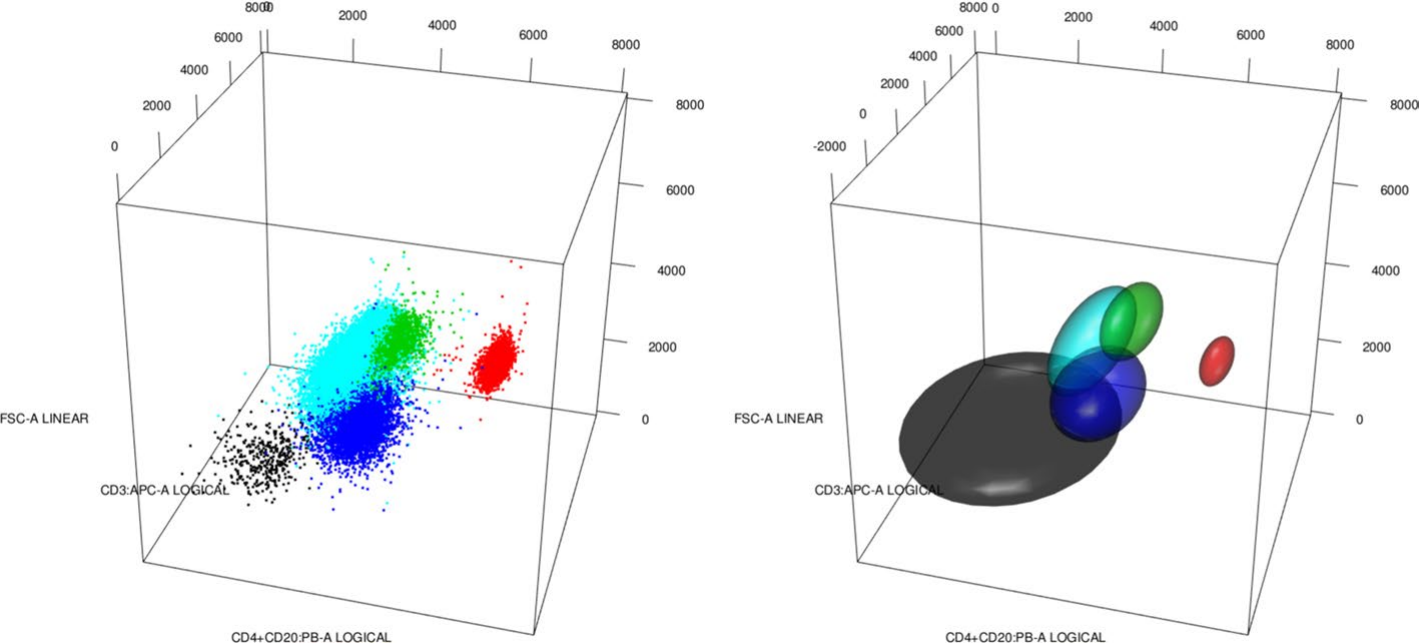
A flow cytometry with five cell types viewed in a three-dimensional projection: left as points with labels; and right as ellipsoids containing 95% probability of multivariate normal distributions. We have Basophils in black, CD4+CD8${-}$ in red, Eosinophils in green, Monocytes in blue and Neutrophils in cyan

We provide an example of the different descriptions in Fig. 1. We have five cell types, hence $L^1=\{Basophils\,(black),\,CD4{+}CD8{-}\,(red),\,Eosinophils\,(green),Monocytes\,(blue),\,Neutrophils\,(Cyan)\}$. We have a three-dimensional projection onto three different markers. We can interpret the image on the left as a plot of the coordinates of every cell with its label, but also as the plot of the group of cells labelled as Basophils (black group), and so on. On the other hand, the plot on the right is a representation of the ellipsoid containing 95% of the probability when we see each cluster as a multivariate normal distribution with mean and covariance corresponding to the empirical mean and covariance. As we see from the plots, all the above descriptions seem to represent the data at hand well and, therefore, all of them could be useful for different applications.

### Obtaining prototypic cytometries: optimalFlowTemplates

Due to the high variability in flow cytometry data we should expect that learning from different elements in the database should produce significantly different results on the classification of a new cytometry $X^T=\{X^T_1,\dots , X^T_{n_T}\}\subset {\mathbb {R}}^d$. Our approach is to search for clusters of existing cytometries in the database. In this way we pursue a notable reduction of variability, thus allowing a good representation of the cytometries in each of these groups through prototypic cytometries. Therefore, using a prototype of a group for learning should produce a similar result for classifying $X^T$ to the one obtained using any other cytometry in the same group.

#### Clustering cytometries

Since gated cytometries can be viewed as partitions and we want to cluster cytometries to reduce variability, we want to do clustering of clusterings, also known as meta-clustering. The methodology we will develop consists of using some meaningful distance between partitions and then applying hierarchical clustering methods. We use hierarchical clustering since it does not rely on a particular distance and therefore it is well suited for handling a variety of distances between objects. This is not the case in many other usual clustering procedures.

As a distance between clusterings we propose to use the *similarity distance* (2), introduced in [15]. It is based on two auxiliary distances. The optimal transport distance between two partitions ${\mathcal {C}}^i$ and ${\mathcal {C}}^j$ is defined as

$$\begin{aligned} d_{OT}({\mathcal {C}}^i,{\mathcal {C}}^{j})=\sum _{k=1}^{k=k_i}\sum _{l=1}^{l=k_{j}}w^*_{kl}d( {\mathcal {C}}^i_k,{\mathcal {C}}^{j}_{l}), \end{aligned}$$

where $d({\mathcal {C}}^i_k,{\mathcal {C}}^{j}_{l})$ is a distance between clusters ${\mathcal {C}}^i_k$ and ${\mathcal {C}}^j_l$. $(w^*_{kl})$ are the solutions of the optimal transport linear program

1 $$\begin{aligned} \begin{array}{ll@{}ll} \text {minimize} &{} \sum \limits _{k=1}^{k=k_i}\sum \limits _{l=1}^{l=k_{j}}w_{kl}d({\mathcal {C}}^i_k, {\mathcal {C}}^{j}_{l})&{}\\ \text {subject to} &{} w_{kl}\ge 0, &{}\quad 1\le k\le k_i,1\le l\le k_j&{}\\ &{} \sum \limits _{l = 1}^{l = k_j}w_{kl} = p^i_k, &{}\quad 1\le k\le k_i&{}\\ &{} \sum \limits _{k = 1}^{k = k_i}w_{kl} = p^j_l, &{}\quad 1\le l\le k_j&{}\\ &{} \sum \limits _{k=1}^{k=k_i}\sum _{l=1}^{l=k_{j}}w_{kl} = 1. \end{array} \end{aligned}$$

$d_{OT}$ measures the cost of the optimal way of transforming one partition into the other. For more detailed explanations on optimal transport see Additional file 1: Notions on optimal transport.

The second auxiliary distance is the naive transport distance, which measures the cost of naively transforming one partition into the other. It is defined as

$$\begin{aligned} d_{NT}({\mathcal {C}}^i,{\mathcal {C}}^j) = \sum _{k=1}^{k_i}\sum _{l=1}^{k_j}p^i_kp^j_ld({\mathcal {C}}^i_k,{\mathcal {C}}^j_l). \end{aligned}$$

The *similarity distance* is defined as the quotient

2 $$\begin{aligned} d_S({\mathcal {C}}^i,{\mathcal {C}}^j) = \frac{d_{OT}({\mathcal {C}}^i,{\mathcal {C}}^j)}{d_{NT}({\mathcal {C}}^i, {\mathcal {C}}^j)}. \end{aligned}$$

We recall that $0\le d_S\le 1$, where $d_S = 0$ means that partitions ${\mathcal {C}}^i,{\mathcal {C}}^j$ are represented by the same clusters with the same weights and $d_S = 1$ means that every cluster in ${\mathcal {C}}^i$ is transported proportionally to every cluster in ${\mathcal {C}}^j$. Therefore, values of $d_S$ close to 0 can be interpreted as high similarity between clusterings, and values of $d_S$ close to 1 can be interpreted as very dissimilar clusterings.

To completely define $d_{S}$, we need to specify a distance between clusters. Our choice is to use the well-known Wasserstein distance (see Additional file 1: Notions on optimal transport) so

3 $$\begin{aligned} d({\mathcal {C}}^i_k,{\mathcal {C}}^{j}_{l}) = {\mathcal {W}}_2(N(m^i_k,S^i_k), N(m^j_l,S^j_l)). \end{aligned}$$

In essence, we are treating clusters as multivariate normal distributions, $N(m^i_k,S^i_k)$ and $N(m^j_l,S^j_l)$, with means and covariances calculated from the clusters. Our choice of the Wasserstein distance is based on the desire to account for the spatial shapes of the clusters and to obtain templates for the groups of cytometries. We stress that all results in this work are also valid when understanding clusters as members of a location-scatter family.

Another interesting measure for cluster difference is the (entropy) regularized Wasserstein distance, ${\mathcal {W}}_\gamma ({\mathcal {C}}^i_k,{\mathcal {C}}^{j}_{l})$, where clusters are understood as empirical distributions. We have written it down in Additional file 1: Notions on optimal transport equation (2). We recall that the entropy-regularized Wasserstein distance is strictly convex and there are efficient solutions based on the Sinkhorn algorithm (see [25]). However, any other dissimilarity measure can be used, and, indeed, several have been used in the context of cluster comparison in FC. For example, the symmetric Kullback–Leibler divergence was used in [13], where

4 $$\begin{aligned} d_{KL}({\mathcal {C}}^i_k,{\mathcal {C}}^{j}_{l})&= \frac{1}{2}\Big (KL(N(m^i_k,S^i_k)\Vert N(m^j_l,S^j_l))\nonumber \\&\quad + KL(N(m^j_l,S^j_l)\Vert N(m^i_k,S^i_k))\Big ), \end{aligned}$$

and the Friedman–Rafsky test statistic was used in [14].

When we see clusters as collections of points, and we have different clusterings of the same data, the Adjusted Rand Index, the Jaccard distance or other similar measures can be used, at the expense of losing spatial information. 
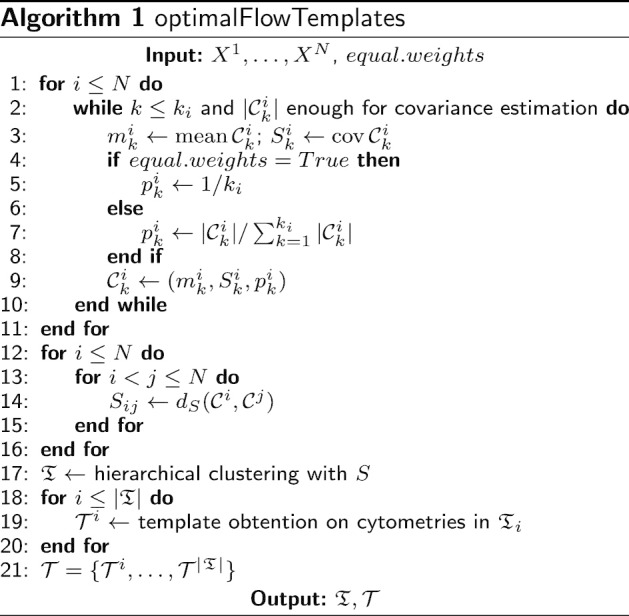


The clustering of cytometries is presented in lines 1–17 in Algorithm 1, resulting in a partition, ${\mathfrak {T}}=\{{\mathfrak {T}}_1,\dots ,{\mathfrak {T}}_{|{\mathfrak {T}}|}\}$, of the input cytometries. Lines 12–16 are concerned with the obtention of a distance matrix *S* that, in line 17, is used to perform hierarchical clustering. Classical agglomerative algorithms can be used, but also density-based algorithms as DBSCAN (see [26]) and HDBSCAN (see [27]).

#### Template obtention through consensus clustering

At this point we have obtained a partition, ${\mathfrak {T}}$, of the collection of cytometries $\{{\mathcal {C}}^j\}_{j=1}^N$. Next, we want to obtain a prototype cytometry, ${\mathcal {T}}^i$, for every group of cytometries, *i*, in the partition ${\mathfrak {T}}$ (lines 18–21 in Algorithm 1). To address this goal, we resort to k-barycenters using Wasserstein distance, which provide a suitable tool for consensus on probability distributions (see [20]). We propose three different methods to obtain a template cytometry from a group of cytometries, that is, to obtain a consensus (ensemble) clustering on flow cytometries. These methods are given in Algorithms 2, 3 and 4. 
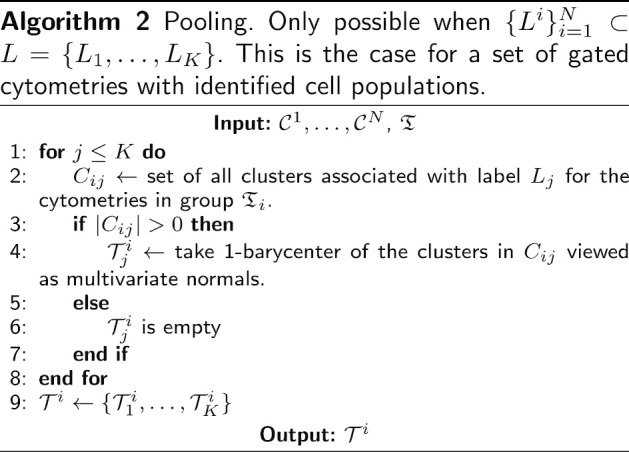

**Fig. 2:**
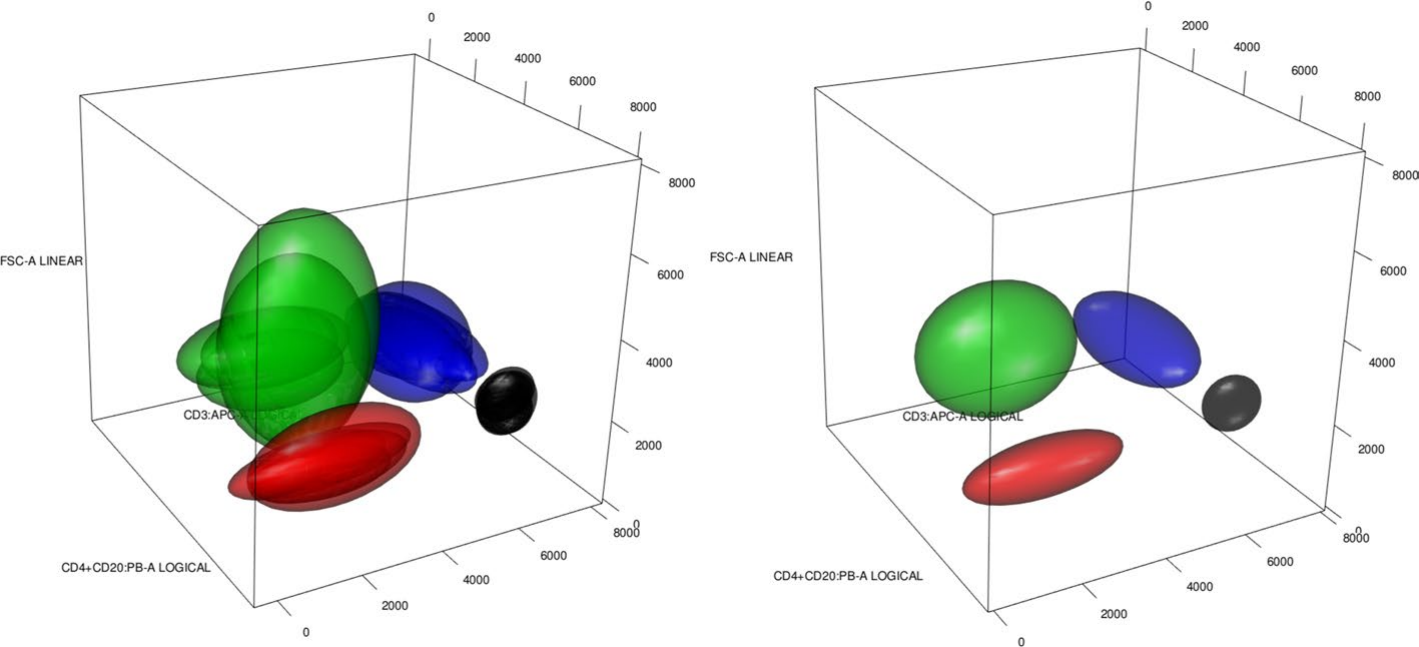
An application of Algorithm 2-Pooling. On the left we have 5 different cytometries, each with 4 different identified cell types given by $\{Monocytes\,(black),\, CD4{+}CD8{-}\,(red),\, Mature\,Sig\,Kappa\,(green),\,TCRgd{-}\,(blue)\}$. On the right we have a prototype cytometry obtained taking the 1-barycenter for each cell type. Ellipsoids contain 95% of the probability of the respective multivariate gaussian distributions

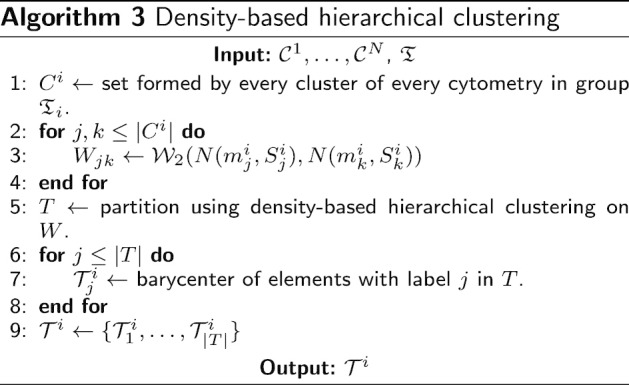



**Fig. 3:**
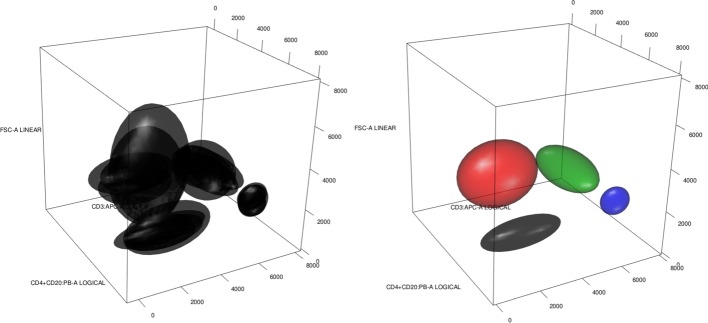
Application of Algorithm 3—densit-based. On the left we have the same 5 cytometries as in Fig. 2, but each cytometry is grouped in clusters without cell types being identified. On the right we have a prototype cytometry obtained taking the denisty based hierarchical clustering approach on the cytometries represented on the left. Ellipsoids contain 95% of the probability of the respective multivariate gaussian distributions

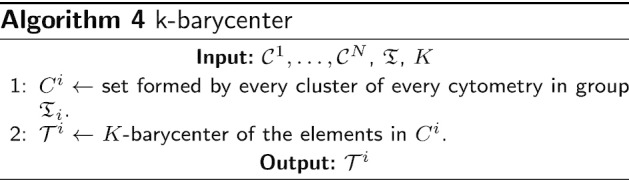



**Fig. 4:**
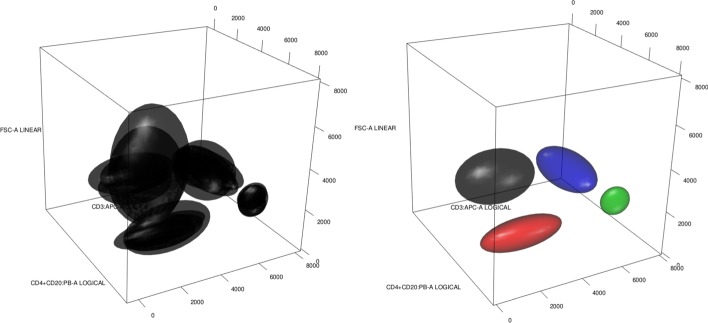
Application of Algorithm 4—4-barycenter. On the left we have the same 5 cytometries as in Fig. 2, but each cytometry is grouped in clusters without cell types being identified. On the right we have a prototype cytometry obtained taking the 4-barycenter of the cytometries represented on the left. Ellipsoids contain 95% of the probability of the respective multivariate gaussian distributions

The intention behind pooling (Algorithm 2), is to take advantage of having groups of similar cytometries and knowing the actual cell types in them. A prototype of a cell type is obtained through a (1-)barycenter—a consensus representation—of the multivariate distributions that represent the same cell type in the cytometries that are members of the same group in ${\mathfrak {T}}$. A prototype cytometry is the collection of prototypes of each cell type. This can be seen in Fig. 2. On the left-hand side, we have 5 different cytometries, each with 4 different cell types, hence $L = \{Monocytes\,(black),\, CD4{+}CD8{-}\,(red),\, Mature\,Sig\,Kappa\,(green),\,TCRgd{-}\,(blue)\}$. Since the cell types are known, we take all the black ellipsoids of the left plot, representing the different normal distributions, and obtain the black ellipsoid on the right plot, the barycenter of the group of normal distributions, as a consensus element for Monocytes. Doing this for every cell type gives us the prototype cytometry represented on the right of Fig. 2.

However, our templates could be obtained even when we have gated cytometries but without identified cell types. This could be the case when unsupervised gating is used to obtain a database of gated cytometries. Density-based hierarchical clustering (Algorithm 3) and k-barycenter (Algorithm 4) are based on the idea that clusters that are close in Wasserstein distance should be understood as representing the same, although we may not know which, cell type. When using k-barycenters we must specify the number of cell types, *K*, that we want for the artificial cytometry. However, when using density-based hierarchical clustering as HDBSCAN or DBSCAN the selection of the number of cell types for the prototype cytometry is automatic. Recall that both k-barycenters, through trimming, and density-based hierarchical clustering, are robust clustering procedures.

In Figs. 3 and 4 we have a representation of how Algorithms 3 and 4 work. Since we do not have cell type information for the 5 gated cytometries, we obtain the plot that can be seen on the left of Figs. 3 and 4. However, the absence of this information can be mitigated using the spatial information, which clearly shows a group structure between the ellipsoids. We use density-based hierarchical clustering and k-barycenters respectively, to try to capture this spatial information. As a result, we obtain the template cytometries on the right side of Figs. 3 and 4. Clearly, we see that the templates represent well the real cell types behind the cytometries (compare with Fig. 2), although we still do not know the cell types corresponding to each ellipsoid. This could be achieved using expert information or matching populations.

### Supervised classification: optimalFlowClassification

Now, our goal is to assign cell types to a new cytometry $X^T$, using the information given in a database of gated cytometries $\{{\mathcal {C}}^i\}_{i=1}^N$. The different sources of variability, mainly those of a technical nature and those which are properly due to different cohorts present in the database, advise to search for different cytometric structures. Hence, we should assign $X^T$ to the group of cytometries that is more similar to it and then use supervised techniques. Indeed, this is the purpose of optimalFlowClassification, as shown in Algorithm 5. As an input we apply optimalFlowTemplates to the database $\{{\mathcal {C}}^i\}_{i=1}^N$ to obtain the partition ${\mathfrak {T}}$ and the templates ${\mathcal {T}}$. 
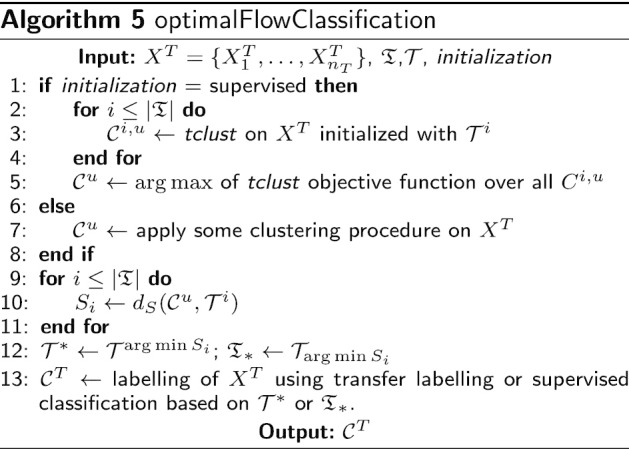


Lines 1–5 in Algorithm 5 are dedicated to finding an unsupervised partition of the new cytometry $X^T$ using as initialization for *tclust* the prototypes of the database. Initializing with the database entries attempts to use the available information optimally. Hence, if $X^T$ is similar to some of the cytometries in the database, appropriate initialization should be advantageous. However, some other suitable unsupervised initializations can be used, such as the ones proposed in FLOCK, flowPeaks or flowMeans. We need to cluster $X^T$ to compare it with the template cytometries.

Notice that *tclust* [21], is a more sophisticated version of k-means, allowing ellipsoidal clusters with different sizes and shapes. Like k-means, this robust model-based clustering procedure needs an initialization and its behaviour improves notably if that initialization is well suited. Nonetheless, it is possible to use any other unsupervised procedure that allows an initialization with a clustering defined by probability distributions. For example, this is the case for the popular *mclust* [28, 29], a finite Gaussian mixture model-based clustering based on an EM-algorithm.

*tclust* searches for a partition $\{{\mathcal {C}}_0,\dots , {\mathcal {C}}_k\}$ of $X = \{X_1,\dots , X_n\}$, with $|{\mathcal {C}}_0|=\lceil n\alpha \rceil$, vectors $m_j$, positive definite matrices $S_j$ and weights $p_j\in [0,1]$ that approximately maximize the pseudo-likelihood

5 $$\begin{aligned} \sum _{j=1}^{k}\sum _{i\in {\mathcal {C}}_j}\log \left( p_j\varphi (X_i;m_j, S_j)\right) , \end{aligned}$$

under restrictions over the scatter matrices $S_j$. By $\varphi (\cdot ;m_j, S_j)$ we denote the density function of the multivariate normal $N(m_j, S_j)$. ${\mathcal {C}}_0$ is the cluster of trimmed observations, where the trimming level is $\alpha$.

The details of the algorithm can be found in [30]. For us it is relevant to recall only the initialization step, i.e., to provide an initial $\theta ^0=(p^0_1,\dots , p^0_k,m_1^0, \dots , m_k^0, S_1^0,\dots , S_k^0)$. Subsequently, we only need a set of weights with corresponding means and covariances to initialize *tclust*.

We favour the use of *tclust* over k-means since it allows for non-spherical clusters and for trimming, making partitions more robust to outliers and even bridge-points. However, our procedure is compatible with any other way of obtaining a partition of the data, which we reflect in lines 6–8. In our experiments we have used flowMeans as an alternative to *tclust*.

In lines 9–12 we look to assign $X^T$, using the clustering ${\mathcal {C}}^u$, produced in the previous step, to the template that is closest in similarity distance to ${\mathcal {C}}^u$. With this we hope to use only the most relevant information of the database, summarized in ${\mathcal {T}}^*$ and ${\mathfrak {T}}_*$.

The last step in Algorithm 5, line 13, assigns cell types to $X^T$. To do this we have several options. We can try to relabel ${\mathcal {C}}^u$ in an optimal way using ${\mathcal {T}}^*$ or ${\mathfrak {T}}_{*}$, i.e., do label transfer. Alternatively, we can use ${\mathcal {T}}^*$ to do Quadratic Discriminant Analysis (QDA). Another possibility is to find the most similar partition in similarity distance (2) from ${\mathfrak {T}}_{*}$ to ${\mathcal {C}}^u$ and use it to do QDA or random forest classification.

For supervised classification we use standard tools, random forest and QDA. However, other methods can be used in a straightforward fashion. We stress that when using QDA and ${\mathcal {T}}^*$ we are using non-linear multidimensional gating regions obtained from ${\mathcal {T}}^*$ to classify $X^T$. This can be taught as an extension of the method presented in [11] where only linear one-dimensional regions are used. Another interesting fact is that the use of $d_S$ allows us to select the most similar real cytometry to $C^u$, resulting in supervised tools being more effective.

The problem of relabelling a clustering ${\mathcal {C}}^j$ with respect to another clustering ${\mathcal {C}}^i$ is usually stated as a weighted bipartite matching problem, where weights are related to the similarity between clusters in the two partitions. This problem can be solved by the Hungarian method [31] or generalized edge cover (see [13]), for example.

Additionally, we introduce an approach to obtain a fuzzy relabelling based on solving the optimal transport linear program associated to (1). The solution, $(w_{kl}^*)$, is the base for this fuzzy relabelling. We define the score of cluster *l* in ${\mathcal {C}}^j$ to come from cluster *k* in ${\mathcal {C}}^i$ as $s^l_k=w^*_{kl}/p^j_l$. In words, $s^l_k$ is the proportion of probability coming from cluster *k*, with respect to the probability in cluster *l*, that arrives at cluster *l*. Clearly, $0\le s^l_k\le 1$, and higher scores indicate more evidence that clusters *k* and *l* represent the same cluster. A fuzzy relabelling for cluster *l* in ${\mathcal {C}}^j$ is the collection of all the scores $s^l = \{s^l_1,\dots ,s^l_{|{\mathcal {C}}^i|}\}$. A variation of the previous score is ${\tilde{s}}^l_k = s^l_k*w^*_{kl}/p^i_k$, where we are weighting by the proportion of cluster *k* that goes to cluster *l*, with respect to the probability contained in cluster *k*. In this way we down-weight the effect of a small proportion of a big cluster with respect to a big proportion of a small cluster arriving to *l*. From these fuzzy relabellings a hard relabelling can be obtained easily.

Again, a suitable distance between clusters can be the Wasserstein distance as in (3), which is computationally very efficient. However, another possibility is to use

6 $$\begin{aligned} d({\mathcal {C}}^i_k,{\mathcal {C}}^j_l) = \frac{1}{|{\mathcal {C}}^i_k||{\mathcal {C}}^j_l|}\sum _{x\in {\mathcal {C}}^i_k}\sum _{y\in {\mathcal {C}}^j_l}\Vert x-y\Vert ^2 \end{aligned}$$

which, unlike the Wasserstein distance, allows the labelling of small clusters in ${\mathcal {C}}^j$, but does so at the price of using sub-sampling to compare bigger clusters (for example, more than 10,000 points).

## Results

In this section we present several experiments and comparisons of our methods with other state-of-the-art procedures on two real datasets.

### Data

The first dataset is formed by cytometries obtained following the Euroflow protocols using a BD FACSCanto flow cytometer in four different international centres. The size of the cytometry datasets varies from 50,000 to 300,000 cells. The samples are from adult male and female individuals, from a variety of age groups and with different conditions (see Table 1). Thus, there is biological variability, since there are different individuals with different conditions, ages, and other different characteristics. Moreover, we have technical variability since we have different centres, different dates of measurement and different incubation times.

The dataset contains 40 gated cytometries, ${\mathcal {C}}=\{{\mathcal {C}}^{1,h}, {\mathcal {C}}^{2,h}, {\mathcal {C}}^{3,h}, {\mathcal {C}}^{4,h}, {\mathcal {C}}^{5,h}, {\mathcal {C}}^{6,h}, {\mathcal {C}}^{7,h},{\mathcal {C}}^{8,h}, {\mathcal {C}}^{9,h}, {\mathcal {C}}^{10,h},$ ${\mathcal {C}}^{11,h}, {\mathcal {C}}^{12,h}, {\mathcal {C}}^{13,h}, {\mathcal {C}}^{14,h}, {\mathcal {C}}^{15,h}, {\mathcal {C}}^{16,h},{\mathcal {C}}^{17,h}, {\mathcal {C}}^{18,h}, {\mathcal {C}}^{19,h},$ ${\mathcal {C}}^{20,h}, {\mathcal {C}}^{21,h},{\mathcal {C}}^{22,s}, {\mathcal {C}}^{23,s}, {\mathcal {C}}^{24,s}, {\mathcal {C}}^{25,s}, {\mathcal {C}}^{26,s}, {\mathcal {C}}^{27,s}, {\mathcal {C}}^{28,s},$ ${\mathcal {C}}^{29,h}, {\mathcal {C}}^{30,h}, {\mathcal {C}}^{31,h}, {\mathcal {C}}^{32,h}, {\mathcal {C}}^{33,h}, {\mathcal {C}}^{34,s},{\mathcal {C}}^{35,s}, {\mathcal {C}}^{36,h}, {\mathcal {C}}^{37,h},$ ${\mathcal {C}}^{38,h}, {\mathcal {C}}^{39,h}, {\mathcal {C}}^{40,h}\}$, where the super index *s* means sick and super index *h* means healthy. Complementary information about the cytometries can be found in Table 1. We split them in a learning set, $\mathcal {DB}=\{{\mathcal {C}}^{1}, {\mathcal {C}}^{3},{\mathcal {C}}^{4},{\mathcal {C}}^{6},{\mathcal {C}}^{8},{\mathcal {C}}^{10},{\mathcal {C}}^{11},{\mathcal {C}}^{12},{\mathcal {C}}^{13},{\mathcal {C}}^{16},{\mathcal {C}}^{19},{\mathcal {C}}^{20},{\mathcal {C}}^{21},$ ${\mathcal {C}}^{22}, {\mathcal {C}}^{23},{\mathcal {C}}^{24},{\mathcal {C}}^{25},{\mathcal {C}}^{28},{\mathcal {C}}^{30},{\mathcal {C}}^{32},{\mathcal {C}}^{33},{\mathcal {C}}^{34},{\mathcal {C}}^{35},{\mathcal {C}}^{36},{\mathcal {C}}^{37},$ ${\mathcal {C}}^{38},{\mathcal {C}}^{39}\}$, and a test set $\mathcal {TS}=\{{\mathcal {C}}^{2},{\mathcal {C}}^{5},{\mathcal {C}}^{7},{\mathcal {C}}^{9},{\mathcal {C}}^{14},{\mathcal {C}}^{15},$ ${\mathcal {C}}^{17},{\mathcal {C}}^{18},{\mathcal {C}}^{26},{\mathcal {C}}^{27},{\mathcal {C}}^{29},{\mathcal {C}}^{31}, {\mathcal {C}}^{40}\}$.

Additionally, in order to explore the behaviour of our procedure in the presence of different sources of variations and make a clear comparison with flowMatch, we use the healthy donor dataset from [32] to further validate our methods. This dataset includes “three sources of variations: (1) technical or instrumental variation among replicates of the same sample, (2) within-subject temporal (day-to-day) variation, and (3) between-subject natural or biological variation”. This dataset is available in the package healthyFlowData in Bioconductor. In our labelling the data correspond to the individuals as follows, A: 1–5, B: 6–10, C: 11–15 and D: 16–20.

**Table 1:** Detailed information about the participants and the measurements for the cytometries used in the experiments

	Center	Final diagnosis	Tested sample	Coagulant	Sex	Age	Incubation period	Flow cytometer
${\mathcal {C}}^{1}$	1	HD	PB	EDTA	M	53	30	BD FACSCanto
${\mathcal {C}}^{2}$	1	HD	PB	EDTA	M	50	30	BD FACSCanto
${\mathcal {C}}^{3}$	1	HD	PB	EDTA	M	61	30	BD FACSCanto
${\mathcal {C}}^{4}$	2	HD	PB	Heparin	M	29	30	BD FACSCanto
${\mathcal {C}}^{5}$	2	HD	PB	Heparin	M	38	30	BD FACSCanto
${\mathcal {C}}^{6}$	2	HD	PB	Heparin	F	27	30	BD FACSCanto
${\mathcal {C}}^{7}$	2	HD	PB	Heparin	F	NA	30	BD FACSCanto
${\mathcal {C}}^{8}$	2	HD	PB	Heparin	M	NA	30	BD FACSCanto
${\mathcal {C}}^{9}$	2	HD	PB	Heparin	F	NA	30	BD FACSCanto
${\mathcal {C}}^{10}$	2	HD	PB	Heparin	F	NA	30	BD FACSCanto
${\mathcal {C}}^{11}$	3	HD	PB	NA	M	34	15	BD FACSCanto
${\mathcal {C}}^{12}$	3	HD	PB	NA	F	33	15	BD FACSCanto
${\mathcal {C}}^{13}$	3	HD	PB	NA	M	32	15	BD FACSCanto
${\mathcal {C}}^{14}$	3	HD	PB	NA	M	33	15	BD FACSCanto
${\mathcal {C}}^{15}$	3	HD	PB	NA	F	35	15	BD FACSCanto
${\mathcal {C}}^{16}$	3	HD	PB	EDTA	NA	Adult	15	BD FACSCanto
${\mathcal {C}}^{17}$	3	HD	PB	EDTA	NA	Adult	15	BD FACSCanto
${\mathcal {C}}^{18}$	3	HD	PB	EDTA	NA	Adult	15	BD FACSCanto
${\mathcal {C}}^{19}$	3	HD	PB	EDTA	NA	Adult	15	BD FACSCanto
${\mathcal {C}}^{20}$	3	HD	PB	EDTA	NA	Adult	15	BD FACSCanto
${\mathcal {C}}^{21}$	NA	HD	NA	NA	NA	NA	NA	BD FACSCanto
${\mathcal {C}}^{22}$	4	MCL	PB	NA	F	82	15	BD FACSCanto
${\mathcal {C}}^{23}$	4	MCL	PB	NA	M	70	15	BD FACSCanto
${\mathcal {C}}^{24}$	4	FL	BM	NA	M	52	15	BD FACSCanto
${\mathcal {C}}^{25}$	4	MCL	BM	NA	M	81	15	BD FACSCanto
${\mathcal {C}}^{26}$	4	LPL	PB	NA	M	67	15	BD FACSCanto
${\mathcal {C}}^{27}$	1	CLL	LN	Other	F	61	30	BD FACSCanto
${\mathcal {C}}^{28}$	1	CLL	LN	Other	F	61	30	BD FACSCanto
${\mathcal {C}}^{29}$	1	HD	PB	EDTA	F	27	30	BD FACSCanto
${\mathcal {C}}^{30}$	1	HD	PB	EDTA	M	54	30	BD FACSCanto
${\mathcal {C}}^{31}$	1	HD	PB	EDTA	M	50	30	BD FACSCanto
${\mathcal {C}}^{32}$	1	HD	PB	EDTA	F	36	30	BD FACSCanto
${\mathcal {C}}^{33}$	1	HD	PB	EDTA	M	74	30	BD FACSCanto
${\mathcal {C}}^{34}$	1	DLBCL	Other	Other	M	65	30	BD FACSCanto
${\mathcal {C}}^{35}$	1	HCL	BM	EDTA	M	40	30	BD FACSCanto
${\mathcal {C}}^{36}$	2	HD	PB	Heparin	M	38	30	BD FACSCanto
${\mathcal {C}}^{37}$	2	HD	PB	Heparin	F	27	30	BD FACSCanto
${\mathcal {C}}^{38}$	2	HD	PB	Heparin	M	NA	30	BD FACSCanto
${\mathcal {C}}^{39}$	2	HD	PB	Heparin	F	NA	30	BD FACSCanto
${\mathcal {C}}^{40}$	3	HD	PB	EDTA	NA	NA	15	BD FACSCanto

Samples come from four international centers labelled as 1–4. Diagnosis abbreviations correspond to: healthy diagnosis, mantle cell lymphoma, follicular lymphoma, lymphoplasmacytic lymphoma, chronic lymphocytic leukemia, diffuse large B-cell lymphoma and hairy cell leukemia. The abbreviations for the type of tested samples correspond to: peripherial blood, bone marrow, lymph node. Coagulant refers to the type of coagulant used for preservation of the sample. The incubation period is measured in minutes

### Measures of performance

We need appropriate methods to measure the performance of the different automated gating procedures that appear in this work. We recall that we use both unsupervised and supervised methods. In this setup an appropriate tool is the *F-measure* statistic which has been used in [1, 3, 9, 10]. With our notation we have

7 $$\begin{aligned}&F({\mathcal {C}}^i,{\mathcal {C}}^j)=\sum _{k=1,\dots ,|{\mathcal {C}}^i|} \frac{|{\mathcal {C}}^i_k|}{M}\max _{l=1,\dots ,|{\mathcal {C}}^j|}F({\mathcal {C}}^i_k, {\mathcal {C}}^j_l), \end{aligned}$$

8 $$\begin{aligned}&F({\mathcal {C}}^i_k,{\mathcal {C}}^j_l) = 2\frac{R({\mathcal {C}}^i_k,{\mathcal {C}}^j_l) P({\mathcal {C}}^i_k,{\mathcal {C}}^j_l)}{R({\mathcal {C}}^i_k,{\mathcal {C}}^j_l) + P({\mathcal {C}}^i_k,{\mathcal {C}}^j_l)}, \end{aligned}$$

9 $$\begin{aligned}&R({\mathcal {C}}^i_k,{\mathcal {C}}^j_l) = \frac{|{\mathcal {C}}^i_k \cap {\mathcal {C}}^j_l|}{|{\mathcal {C}}^i_k|}\quad \text {and} \quad P({\mathcal {C}}^i_k,{\mathcal {C}}^j_l) = \frac{|{\mathcal {C}}^i_k \cap {\mathcal {C}}^j_l|}{|{\mathcal {C}}^j_l|} \end{aligned}$$

with $M= \sum _{k=1,\dots ,|{\mathcal {C}}^i|}|{\mathcal {C}}^i_k|=\sum _{l=1,\dots ,|{\mathcal {C}}^j|}|{\mathcal {C}}^j_l|$. We make the convention $R(\emptyset ,{\mathcal {C}}^j_l) = P({\mathcal {C}}^i_k,\emptyset ) = 1$ and $R({\mathcal {C}}^i_k,\emptyset ) =P(\emptyset ,{\mathcal {C}}^j_l) = 0$. Another appealing measure is the *median F-measure* used in [11] specifically for supervised learning. The formal definition is

10 $$\begin{aligned}&{\tilde{F}}({\mathcal {C}}^i,{\mathcal {C}}^j) =\mathrm {median}\Big \{\big \{F({\mathcal {C}}^i_k,{\mathcal {C}}^j_{k*}):\nonumber \\&k \text { such that } L^i_k=L^j_{k^*}\in L^i\cap L^j\big \}, \{0\}\times |L^i\triangle L^k|\Big \} \end{aligned}$$

where ${\mathcal {C}}^i$ is the ground truth, in our case a manual gating, and ${\mathcal {C}}^j$ is the classification obtained for the same data.

### Clustering cytometries and template obtention

We want to compare different methods for clustering a database. For a state-of-the-art comparison, we use flowMatch [13]. Notice that flowMatch is based on a GEC procedure, a generalization of bipartite matching, where the cost between partitions is given by

**Fig. 5:**
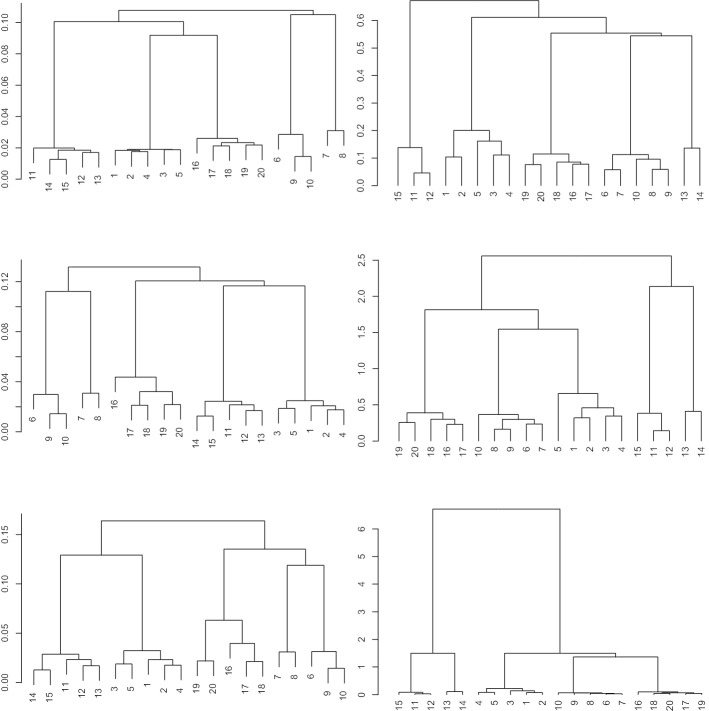
Hierarchical trees for the healthyFlowData. First column: result of optimalFlowTemplates for single linkage (top), average linkage (middle) and complete linkage (bottom). Second column: result of flowMatch with Euclidean distance (top), Mahalanobis distance (middle) and Kullback–Libler divergence (bottom)

**Fig. 6:**
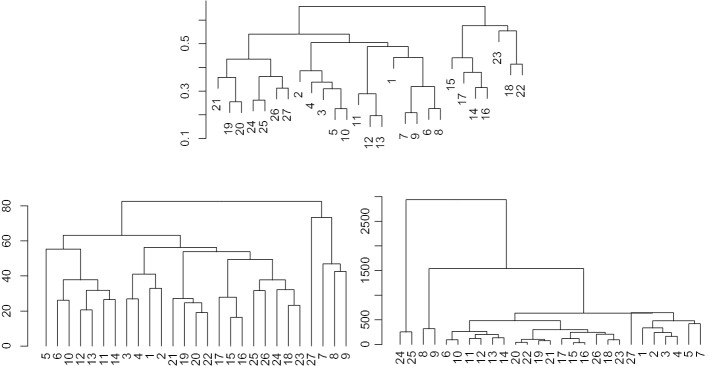
Hierarchical trees for $\mathcal {DB}$. First row: result of optimalFlowTemplates for complete linkage. Second row: result of flowMatch with Mahalanobis distance (left) and Kullback–Libler divergence (right)

11 $$\begin{aligned} d({\mathcal {C}}^i,{\mathcal {C}}^j)=\frac{1}{k_ik_j}\sum _{k=1}^{k_i} \sum _{l=1}^{k_j}d_{KL}({\mathcal {C}}^i_k,{\mathcal {C}}^j_l), \end{aligned}$$

where $d_{KL}$ is as in (4), or

12 $$\begin{aligned} d({\mathcal {C}}^i,{\mathcal {C}}^j)=\frac{1}{k_ik_j}\sum _{k=1}^{k_i} \sum _{l=1}^{k_j}d_{Mahalanobis}(N(m^i_k, S^i_k),N(m^j_l, S^j_l)), \end{aligned}$$

where $d_{Mahalanobis}$ is the well-known Mahalanobis distance between multivariate normals.

For a comparison with different variability sources and a clear ground truth we cluster the healtyFlowData and present the results in Fig. 5. The left column presents the results when we use optimalFlowTemplates, or equivalently when we use similarity distance as a distance between cytometries. The right column presents the results when using flowMatch and distances between cytometries given by (12) and (11) and an appropriate Euclidean distance-based modification. In Fig. 6 we present a similar procedure but for the training set $\mathcal {DB}$ of our first dataset.

In Table 2 we see two different clusterings obtained when using optimalFlowTemplates. We recall that HDBSCAN automatically selects the number of clusters. We also stress that the clustering obtained from complete linkage comes from the appropriate pruning of the tree shown top in Fig. 6.

We notice that $\mathcal {DB}$ is relabelled from 1 to 27 as shown in the first row in Table 2 and these are the labels used in Fig. 6. Let us stress that labels $\{14,15,16,17,18,22,23\}$ correspond to the cytometries $\{{\mathcal {C}}^{22}, {\mathcal {C}}^{23}, {\mathcal {C}}^{24},{\mathcal {C}}^{25},{\mathcal {C}}^{28}, {\mathcal {C}}^{34},{\mathcal {C}}^{35}\}$ that represent individuals with cancer.

**Table 2:** Clustering of the cytometries in $\mathcal {DB}$ obtained using optimalFlowTemplates with complete linkage hierarchical clustering looking for 7 clusters and using HDBSCAN

ID	Cytometry	Cluster	
		Comp.-Link.	HDBSCAN
1	${\mathcal {C}}^{1}$	1	1
2	${\mathcal {C}}^{3}$	2	6
3	${\mathcal {C}}^{4}$	2	6
4	${\mathcal {C}}^{6}$	2	6
5	${\mathcal {C}}^{8}$	2	6
6	${\mathcal {C}}^{10}$	1	8
7	${\mathcal {C}}^{11}$	1	9
8	${\mathcal {C}}^{12}$	1	8
9	${\mathcal {C}}^{13}$	1	9
10	${\mathcal {C}}^{16}$	3	6
11	${\mathcal {C}}^{19}$	3	7
12	${\mathcal {C}}^{20}$	3	7
13	${\mathcal {C}}^{21}$	4	7
14	${\mathcal {C}}^{22}$	4	2
15	${\mathcal {C}}^{23}$	4	2
16	${\mathcal {C}}^{24}$	4	2
17	${\mathcal {C}}^{25}$	4	2
18	${\mathcal {C}}^{28}$	5	3
19	${\mathcal {C}}^{30}$	6	4
20	${\mathcal {C}}^{32}$	6	4
21	${\mathcal {C}}^{33}$	6	4
22	${\mathcal {C}}^{34}$	5	3
23	${\mathcal {C}}^{35}$	7	3
24	${\mathcal {C}}^{36}$	6	5
25	${\mathcal {C}}^{37}$	6	5
26	${\mathcal {C}}^{38}$	6	5
27	${\mathcal {C}}^{39}$	6	5

### Gating and classification

We will use the results of optimalFlowTemplates applied to the database $\mathcal {DB}$, introduced in the previous section, as entries to optimalFlowClassification to automatically perform gating in $\mathcal {TS}$. For the cytometries in $\mathcal {TS}$, we also perform an unsupervised gating given by flowMeans. Results are shown in the first and last columns of Table 3.

We also compare our methods with a state-of-the-art supervised procedure. In this case we will use DeepCyTOF, with some bug corrections and some adaptations to our setting of the Github version, implemented in Python with *tensorflow* 0.12 and *keras* 1.2.2. To use DeepCyTOF we need cytometries with the same number and types of cells, so we use a data set $\mathcal {TS}' = \{{\mathcal {C}}^{2'},{\mathcal {C}}^{5'},{\mathcal {C}}^{7'},{\mathcal {C}}^{14'},{\mathcal {C}}^{15'},{\mathcal {C}}^{17'},{\mathcal {C}}^{18'}\}$, where we have extracted the common groups from the original cytometries. Hence, comparisons in Table 3 are biased in favour of DeepCyTOF, since for optimalFlowClassification we use the original complete cytometries. We want to emphasize that DeepCyTOF only uses the supervised information from one of the cytometries in $\mathcal {TS}'$ to classify all the others. This is shown in Table 3 in italic. Results of DeepCyTOF are provided, with domain adaptation and without de-noising, since all entries are classified, in column 2 of Table 3.

**Table 3:** Table of F-measure statistics as given by (7), where we use the manual gating as the ground truth

	flowMeans	DeepCyTOF	optimalFlowTemplates + DeepCyTOF	optimalFlowTemplates + optimalFlowClassification
${\mathcal {C}}^2$	0.8988	0.9546	*0.9736*	0.9610
${\mathcal {C}}^5$	0.8977	0.9161	0.9196	0.9587
${\mathcal {C}}^7$	0.9508	0.7514	*0.9769*	0.9768
${\mathcal {C}}^9$	0.8936			0.9172
${\mathcal {C}}^{14}$	0.9004	*0.9838*	0.9530	0.9066
${\mathcal {C}}^{15}$	0.8974	0.9408	0.9352	0.9556
${\mathcal {C}}^{17}$	0.9405	0.7847	*0.9810*	0.9848
${\mathcal {C}}^{18}$	0.9004	0.7837	0.9796	0.9849
${\mathcal {C}}^{26}$	0.9024			0.9313
${\mathcal {C}}^{27}$	0.8645			0.9306
${\mathcal {C}}^{29}$	0.9475			0.9744
${\mathcal {C}}^{31}$	0.9290			0.9656
${\mathcal {C}}^{40}$	0.9330			0.9538

First column: results of the unsupervised gating procedure flowMeans on $\mathcal {TS}$. Second column: results of the supervised procedure DeepCyTOF on $\mathcal {TS}'$. Third column, results of DeepCyTOF on the clusters $\mathcal {TS}'_1$, $\mathcal {TS}'_2$ and $\mathcal {TS}'_3$ produced by optimalFlowTemplates. Forth column: results of our supervised procedure optimalFlowTemplates + optimalFlowClassification on $\mathcal {TS}$. In underline we have the best performance according to the F-measure

**Fig. 7:**
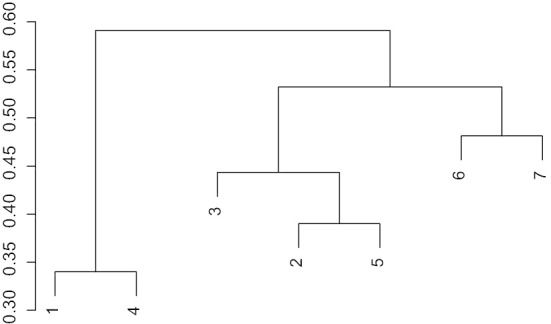
Hierarchical tree obtained by using optimalFlowTemplates with complete linkage on the databse $\mathcal {TS}'$ after gating each cytometry with the unsupervised procedure flowMeans

We see that DeepCyTOF performs rather poorly for $\{{\mathcal {C}}^{7'},{\mathcal {C}}^{17'},{\mathcal {C}}^{18'}\}$ due to the high variability of the cytometries in $\mathcal {TS}'$, which cannot be accommodated by DeepCyTOF’s domain adaptation procedure. Hence, if we were able to reduce this variability, DeepCyTOF should give better results. Indeed, if we use flowMeans to gate the cytometries in $\mathcal {TS}'$, and then we use optimalFlowTemplates, we obtain the hierarchical tree presented in Fig. 7. It suggests splitting $\mathcal {TS}'$ into $\mathcal {TS}'_1=\{{\mathcal {C}}^{2'},{\mathcal {C}}^{14'}\}$, $\mathcal {TS}'_2=\{{\mathcal {C}}^{5'},{\mathcal {C}}^{7'},{\mathcal {C}}^{15'}\}$ and $\mathcal {TS}'_3=\{{\mathcal {C}}^{17'},{\mathcal {C}}^{18'}\}$. We highlight that until now we have not used any supervised information. Applying DeepCyTOF to $\mathcal {TS}'_1$, $\mathcal {TS}'_2$ and $\mathcal {TS}'_3$ we obtain the results in column 3 of Table 3. Again, in italic we have the cytometry which DeepCyTOF uses for learning in each group.

**Table 4:** Parameters and performance (as measured by (10) and manual gating as ground truth) of the best results obtained by optimalFlowTemplates + optimalFlowClassification on $\mathcal {TS}$

	${\mathcal {C}}^2$	${\mathcal {C}}^{5}$	${\mathcal {C}}^{7}$
*Median F-measure*	0.9441931	0.8530806	0.957045
*Database Clustering*	Complete linkage	HDBSCAN	Complete linkage
*Template Formation*	Pooling	Pooling	HDBSCAN
*Assigned Cluster*	1	6	2
*Sample Clustering*	tclust	tclust	tclust
*Supervised Classification*	QDA	QDA from template	Random forest
*Assigned Cytometry*	${\mathcal {C}}^{1}$		${\mathcal {C}}^{8}$

	${\mathcal {C}}^{9}$	${\mathcal {C}}^{14}$	${\mathcal {C}}^{15}$
*Median F-measure*	0.9458429	0.9254252	0.8807339
*Database Clustering*	HDBSCAN	HDBSCAN	HDBSCAN
*Template Formation*	Pooling	k-barycenter	k-barycenter
*Assigned Cluster*	9	1	1
*Sample Clustering*	tclust	tclust	tclust
*Supervised Classification*	QDA	Label transfer with (6)	Random forest
*Assigned Cytometry*	${\mathcal {C}}^{13}$		${\mathcal {C}}^{1}$

	${\mathcal {C}}^{17}$	${\mathcal {C}}^{18}$	${\mathcal {C}}^{26}$
*Median F-measure*	0.9679446	0.9575489	0.8316279
*Database Clustering*	HDBSCAN	HDBSCAN	Complete linkage
*Template Formation*	HDBSCAN	HDBSCAN	HDBSCAN
*Assigned Cluster*	7	7	4
*Sample Clustering*	tclust	flowMeans	tclust
*Supervised Classification*	Random forest	Random forest	Random forest
*Assigned Cytometry*	${\mathcal {C}}^{20}$	${\mathcal {C}}^{20}$	${\mathcal {C}}^{24}$

	${\mathcal {C}}^{27}$	${\mathcal {C}}^{29}$	${\mathcal {C}}^{31}$
*Median F-measure*	0.9312977	0.9259644	0.931515
*Database Clustering*	Complete linkage	Complete linkage	HDBSCAN
*Template Formation*	Pooling	k-barycenter	Pooling
*Assigned Cluster*	5	6	4
*Sample Clustering*	tclust	flowMeans	tclust
*Supervised Classification*	Random forest	Random forest	QDA from template
*Assigned Cytometry*	${\mathcal {C}}^{28}$	${\mathcal {C}}^{33}$	

	$${\mathcal {C}}^{40}$$		
*Median F-measure*	0.8240522		
*Database Clustering*	Complete linkage		
*Template Formation*	Pooling		
*Assigned Cluster*	6		
*Sample Clustering*	tclust		
*Supervised Classification*	Random forest		
*Assigned Cytometry*	${\mathcal {C}}^{30}$		

*Database Clustering* refers to the clustering method used in line 17 in Algorithm 1. *Template Formation* refers to the method used in line 19 in Algorithm 1. *Assigned Cluster* refers to the label of the cluster as given in Table 2 to which the new cytometry is assigned. *Sample Clustering* refers to how we obtain ${\mathcal {C}}^u$ in Algorithm 5. *Supervised Classification* refers to the method used in line 13 in Algorithm 5. *Assigned Cytometry* refers to the optimal cytometry in the respective cluster that is used for learning (when applicable)

In Table 4, we present the best results, as measured by median F-measure (10), of applying optimalFlowTemplates + optimalFlowClassification to $\mathcal {TS}$. For instance, for ${\mathcal {C}}^{27}$, a 61-year-old female with Chronic Lymphocytic Leukemia (see Table 1), we have obtained a very satisfactory median F-measure of 0.9313. This value has been obtained using optimalFlowTemplate followed by optimalFlowClassification. What follows is an extended explanation of how to understand the entries of Table 4.

For optimalFlowTemplates we have used complete linkage to cluster the cytometries in $\mathcal {DB}$ (third column in Table 2) and pooling to obtain the templates. This information is provided in the entries *Database Clustering* and *Template Formation* in Table 4 corresponding to ${\mathcal {C}}^{27}$ (in red). For optimalFlowClassification, we have clustered ${\mathcal {C}}^{27}$ (without using the manual gating information) with *tclust*, as indicated in lines 1–5 in Algorithm 5, obtaining ${\mathcal {C}}^u$. This is reflected in the entry *Sample Clustering*. Then, we assigned ${\mathcal {C}}^{u}$ to the cluster, $\{{\mathcal {C}}^{28},{\mathcal {C}}^{34}\}$, labelled as 5, in column 3 of Table 2. This is shown in the entry *Assigned Cluster*. In order to use random forests for classification, as reflected in the entry *Supervised Classification*, we have assigned ${\mathcal {C}}^u$ to the closest cytometry in similarity distance in the cluster, i.e., the assigned cytometry for learning is ${\mathcal {C}}^{28}$ as reflected in the entry *Assigned Cytometry*.

**Table 5:** F-measure values (as in (8)) for each cell type for ${\mathcal {C}}^{17}$ and ${\mathcal {C}}^{27}$ which are used to obtain the respective median F-measure value in Table 4

	${\mathcal {C}}^{17}$			${\mathcal {C}}^{27}$		
	F-measure	Precision	Recall	F-measure	Precision	Recall
Abnormal Sig Kappa				0.9697	0.9478	0.9925
CD4+CD8${-}$	0.9973	0.9982	0.9965	0.9828	0.9796	0.9859
CD8+CD4${-}$	0.9960	0.9960	0.9960	0.9769	0.9835	0.9704
Neutrophils	0.9968	0.9959	0.9978	0.9421	0.9092	0.9775
Debris/Doublets	0.9692	0.9818	0.9570	0.7704	0.9111	0.6673
Monocytes	0.9679	0.9571	0.9791	0.8419	0.8475	0.8364
Mature Sig Lambda	0.9897	0.9939	0.9856	0.9561	0.9864	0.9277
Mature Sig Kappa	0.9923	0.9866	0.9981	0.9421	0.9421	0.9421
TCRgd${-}$	0.9810	0.9777	0.9843	0.5549	0.5698	0.5408
TCRgd${-}$	0.9403	0.9145	0.9677	0.8634	0.8195	0.9122
CD4+CD8dim	0.9452	0.9504	0.9401	0.5899	0.7111	0.5039
NK cells				0.9313	0.9433	0.9196
Myeloid Cells				0.8321	0.9489	0.7409
CD56dim	0.9827	0.9683	0.9975			
Eosinophils	0.9722	0.9713	0.9732			
Monocytoid DC	0.9563	0.9713	0.9417			
Basophils	0.9123	0.9877	0.8476			
Neutrophils (U.S.)	0.7632	0.7106	0.8242			
Myeloid DC	0.8908	0.8413	0.9464			
CD56bright	0.8785	0.9860	0.7921			
Plasmocytoid DC	0.7790	0.8011	0.7581			
Plasma Cells	0.9677	1.0000	0.9375			

Recall and precision are defined in (9)

## Discussion

From the results shown in Section Clustering cytometries and template obtention, particularly in Fig. 5, we see that optimalFlowTemplates produces trees very similar to the ones given by flowMatch and that it also captures accurately the ground truth. We see that both procedures identify the data coming from the same individuals in 2 of the 3 shown cases.

As shown in Fig. 6, it seems that optimalFlowTemplates captures the difference between healthy and sick individuals accurately in the $\mathcal {DB}$ example. This comes from the fact that clusters of sick individuals merge other clusters high in the tree. Hence, producing clusters from cuts high in the tree will form separate clusters for healthy and sick individuals. However, we see that this is not entirely the case for flowMatch. Therefore, in this case, optimalFlowTemplates offers an advantage over flowMeans when we want to produce templates that will be used for classifying a new cytometry.

Some additional facts should be stated: first, the similarity distance is independent of parameters, something that is not the case for the generalized edge cover distance used in flowMatch. Second, optimalFlowTemplates produces templates only at one stage, once the number of clusters is determined, while flowMatch produces templates at every stage of the hierarchical clustering procedure. Third, optimalFlowTemplates uses a similarity distance which is bound between 0 and 1 and has a clear meaning. However, no such bounds are available for flowMatch. Fourth, flowMatch could be adapted to use the Wasserstein distance between cell types, but this is not implemented in the Bioconductor package that we used to make our comparisons.

We also notice that optimalFlowTemplates can capture differences within the groups of healthy and sick individuals. This is seen in Fig. 5. Therefore, it seems to be capturing additional biological and/or technical variability and not just the one provided by the distinction between healthy and sick. This can be clearly seen in Table 2, where we have several clusters for healthy individuals and several clusters for sick individuals. Hence, we can infer that the similarity distance is sensitive enough to differentiate between cytometries, and therefore that it is a suitable distance for comparing them.

Raw FC data are usually processed via different transformations to produce data more suitable for analysis. This raises two relevant questions: first, what are the effects of the transformations on the hierarchies and the templates that we obtain?; and second, are the templates and hierarchies invariant under transformations? We thank the referee that suggested us these topics.

To address the first question, we will assume that all raw cytometries will be submitted to the same type of transformation. In the setting we have presented, which is mainly model-based, the more the data look like mixtures of a location-scale distribution, the better the performance will be, and the more realistic the artificial templates will seem. On the other hand, if we choose a cost in the similarity distance (2) that is non-parametric, such transformations may not be needed, although they may prove to be helpful. However, templates may be more unrealistic since the location-scale approximation may not be well suited. We expect to address production of templates in a non-parametric fashion in future work.

The hierarchies and templates we obtain, as well as the ones obtained using procedures as flowMatch and QFMatch, are not transformation invariant. Similar transformations will produce similar hierarchies and similar templates. Therefore, from a purely mathematical point of view, there is no true or correct hierarchy. However, from a practical point of view, transformations that allow to produce hierarchies and templates that capture more relevant information for the problem at hand should be preferred.

With respect to the results shown in Section Gating and classification, there are several interesting implications. Firstly, as expected, our supervised method, optimalFlowTemplates + optimalFlowClassification outperforms an unsupervised method such as flowMeans. This is seen in the higher values in each entry of column 3, with respect to column 1, of Table 3. We also see that the F-measures obtained by our procedure are very satisfactory giving a mean value of 0.9539 and a median value of 0.9587 for $\mathcal {TS}$. It is also worth noting that good results are obtained both for healthy individuals and for cancer patients.

Secondly, a comparison with a supervised method, DeepCyTOF, based on neural networks and domain adaptation has been provided. We want to stress that, at least with the implementation provided in Github, we were unable to apply DeepCyTOF to cytometries with different cell types, which limits the applicability of this method. Hence, we had to produce a modified test group given by $\mathcal {TS}'$. This favours DeepCyTOF since our procedure was instead applied to the original cytometries. From column 2 of Table 3, we see that DeepCyTOF works well for cytometries $\{{\mathcal {C}}^{2'},{\mathcal {C}}^{5'},{\mathcal {C}}^{14'},{\mathcal {C}}^{15'}\}$ giving results comparable to our own procedure. However, it does not work as well for cytometries $\{{\mathcal {C}}^{7'}, {\mathcal {C}}^{17'}, {\mathcal {C}}^{18'}\}$. We see that our procedure outperforms DeepCyTOF for every cytometry in $\mathcal {TS}'$, even in the previously mentioned disadvantageous position, except for ${\mathcal {C}}^{14'}$. We stress that DeepCyTOF is using precisely ${\mathcal {C}}^{14'}$ for learning, as indicated in italic in column 2 of Table 3. Therefore, it is using 80% of the data in ${\mathcal {C}}^{14'}$ to calibrate the neural network. Hence, it is natural that DeepCyTOF is the best procedure for this cytometry.

Thirdly, because of the versatility of our procedure optimalFlowTemplates, we can use it to improve the results of DeepCyTOF. In essence, DeepCyTOF’s domain adaptation is not able to account for the high variability in $\mathcal {TS}'$, but this is exactly what optimalFlowTemplate is for. We start by creating a set of gated cytometries by gating in an unsupervised fashion, using flowMeans, the cytometries in $\mathcal {TS}'$. Then we apply optimalFlowTemplates and obtain the tree shown in Fig. 7. This suggests splitting the original group of cytometries into three different clusters $\mathcal {TS}'_1$, $\mathcal {TS}'_2$ and $\mathcal {TS}'_3$. Now, applying DeepCyTOF to each cluster separately, we obtain the results shown in column 3 of Table 3, which show a significant improvement with respect to baseline DeepCyTOF for cytometries $\{{\mathcal {C}}^{7'},{\mathcal {C}}^{17'},{\mathcal {C}}^{18'}\}$ and comparable results for $\{{\mathcal {C}}^{2'}, {\mathcal {C}}^{5'},{\mathcal {C}}^{14'},{\mathcal {C}}^{15'}\}$. Hence, our procedure has indeed helped to improve the performance of DeepCyTOF.

It is important to note that when DeepCyTOF learns in a cytometry, as is the case for ${\mathcal {C}}^{14'}$, and $\{{\mathcal {C}}^{2'},{\mathcal {C}}^{7'},{\mathcal {C}}^{17'}\}$, for columns 2 and 3 of Table 3, respectively, it uses 80% of the sample. This justifies the great result for ${\mathcal {C}}^2$ and ${\mathcal {C}}^7$ in column 3 of Table 3.

Additionally, we want to highlight something that is reflected in Table 4. Cytometries $\{{\mathcal {C}}^{26},{\mathcal {C}}^{27}\}$, taken from individuals with cancer, are assigned to clusters of cytometries of patients with cancer. Therefore, our procedure is correctly assigning sick individuals to clusters of sick individuals. Furthermore, when there is a patient with the same type of cancer in the cluster results are very good. This is the case for ${\mathcal {C}}^{27}$, which is assigned to cluster 5 in the third column of Table 2, where ${\mathcal {C}}^{28}$ also has Chronic Lymphocytic Leukemia. It is also worth noting that healthy individuals are, likewise, assigned to clusters of healthy individuals.

The last thing we want to discuss is the meaning of the median F-measure values, as the ones given in Table 4, which are obtained from values such as the ones shown in Table 5. A high median F-measure value indicates that classification is good along all cell types, regardless of the number of cells in each cell type. This is quite important since often cell types with a small number of cells are very relevant for diagnosis. Indeed, from the values in Table 5 we see that our procedure achieves good performance in almost all cell types.

## Conclusion

In this work we have presented a viable automated supervised gating workflow which is efficient, robust, scalable, and accountable. In particular, we ensure efficiency by using automatically produced clusters of previously gated cytometries capable of capturing information such as sickness and other types of variability. Throughout our experiments, we have shown that our method is robust due to the grouping of cytometries and the automatic assignment of a new one to the most similar group.

Our method does not necessarily require manual gating, so it can be applied to big datasets with only computational cost as a burden, with the added benefit of counteracting the shortcomings of the manual gating approach (namely, human error and the need for experts).

The way in which we approached the problem ensures accountability. Cluster memberships can be screened and, furthermore, synthetic prototypes can be manually gated to check their suitability. Hence even when classification is used with some black-box procedure, we have an accurate understanding of the starting point. Finally, we have shown that our method is versatile, as our workflow can incorporate many previously existing tools in automated flow cytometry gating and it can accommodate many supervised learning procedures.

## Supplementary information

**Additional file 1.** Notions on optimal transport. Supporting information is provided in a PDF file, in which we present a brief overview of the main results on optimal transport used in this work.

## Data Availability

With the user in mind we have developed an R package called optimalFlow that implements our methodology which is available in the Bioconductor repository at https://bioconductor.org/packages/optimalFlow. The data used in this work is freely available for researchers after registration on https://www.EuroFlow.org.

## Abbreviations

BM: bone marrow
CLL: chronic lymphocytic leukimia
DLBCL: diffuse large B-cell lymphoma
FC: flow cytometry
FL: follicular lymphoma
GEC: generalized edge cover
HCL: hairy cell leukimia
HD: healthy diagnosis
LN: lymph node
LPL: lymphoplasmacytic lymphoma
MCL: mantle cell lymphoma
PB: peripherial blood
QDA: quadratic discriminant analysis
QF: quadratic form
