# Supplementary material for: optimalFlow: optimal transport approach to flow cytometry gating and population matching
Source: BMC Bioinformatics. 2020 Oct 27;21:479. doi: 10.1186/s12859-020-03795-w (PMC7590740; doi:10.1186/s12859-020-03795-w)
Supplement: Supplementary file 1 — Additional file 1. Notions on optimal transport. Supporting information is provided in a PDF file, in which we present a brief overview of the main results on optimal transport used in this work. [file 12859_2020_3795_MOESM1_ESM.pdf]

Notions on optimal transport

Following (1), let us take $\mathcal{P}(\Omega)$, the space of probability distributions on Ω. For μ, ν in $\mathcal{P}(\Omega)$, let us define $\Pi(\mu, \nu)$ the set of all probability measures π on $\Omega \times \Omega$ with first marginal μ and second marginal ν. The optimal transport cost between the two measures is defined as

$$C(\mu, \nu) = \inf_{\pi \in \Pi(\mu,\nu)} \int c(x, y) d\pi(x, y) \tag{1}$$

where $c(x, y)$ is the cost of transporting one unit of mass from x to y. A probability π that achieves the minimum in (1) is called an optimal coupling, with an associated random variable (X, Y) that has joint distribution π. When μ and ν are discrete, i.e., $\mu = \sum_{i=1}^{n} p_i \delta_{x_i}$ and $\nu = \sum_{j=1}^{m} q_i \delta_{y_i}$, with $x_i, y_j \in \mathbb{R}^d$, the optimal transport problem can be solved as a linear program (see (2)) where

$$C(\mu, \nu) = \sum_{i=1}^{n} \sum_{j=1}^{m} w_{ij}^* c(x_i, y_j),$$

and (w_{ij}^*) are the solutions of the optimal transport linear program

$$\begin{array}{lll} \text{minimize} & \sum_{i=1}^{n} \sum_{j=1}^{m} w_{ij} c(x_i, y_j) & \\ \text{subject to} & w_{ij} \geq 0, & 1 \leq i \leq n, 1 \leq j \leq m \\ & \sum_{j=1}^{m} w_{ij} = p_i, & 1 \leq i \leq n \\ & \sum_{i=1}^{n} w_{ij} = q_j, & 1 \leq j \leq m \\ & \sum_{i=1}^{n} \sum_{j=1}^{m} w_{ij} = 1. & \end{array}$$

For $(\Omega = \mathbb{R}^d, \|\cdot\|)$, with $\|\cdot\|$ the Euclidean norm, and $p \in [1, \infty)$, the $p-$Wasserstein distance between μ and ν is defined as

$$\begin{aligned} \mathcal{W}_p^p(\mu, \nu) &= \inf_{\pi \in \Pi(\mu,\nu)} \int \|x - y\|^p d\pi(x, y) \\ &= \inf \left\{ E\|X - Y\|^p, \mathcal{L}(X) = \mu, \mathcal{L}(Y) = \nu \right\}, \end{aligned}$$

where $\mathcal{L}(X)$ refers to the law of X.

We present the entropy regularized Wasserstein distance, since it is strictly convex and there are efficient solutions based on the Sinkhorn algorithm (see (3)). For a fixed $\gamma > 0$ the regularized Wasserstein distance is defined as

$$\mathcal{W}_\gamma(\mu, \nu) = \sum_{i=1}^{n} \sum_{j=1}^{m} w_{ij}^* \|x_i - y_j\|^2 + \gamma \sum_{i=1}^{n} \sum_{j=1}^{m} w_{ij}^* \log w_{ij}^*, \tag{2}$$

where (w_{ij}^*) are the solutions of the optimal transport linear program

$$\begin{array}{lll} \text{minimize} & \sum_{i=1}^{n} \sum_{j=1}^{m} w_{ij} \|x_i - y_j\|^2 + \gamma \sum_{i=1}^{n} \sum_{j=1}^{m} w_{ij} \log w_{ij} & \\ \text{subject to} & w_{ij} \geq 0, & 1 \leq i \leq n, 1 \leq j \leq m \\ & \sum_{j=1}^{m} w_{ij} = p_i, & 1 \leq i \leq n \\ & \sum_{i=1}^{n} w_{ij} = q_j, & 1 \leq j \leq m \\ & \sum_{i=1}^{n} \sum_{j=1}^{m} w_{ij} = 1. & \end{array}$$

Let us denote $\mathcal{P}_2(\mathbb{R}^d)$ the set of probability measures on $\mathbb{R}^d$ with finite second moment and let us consider $\mathcal{W}_2(\mu, \nu)$ for $\mu, \nu \in \mathcal{P}(\mathbb{R}^d)$. In (4) the notions of k-barycenter and trimmed k-barycenter were introduced, building on the concept of Wasserstein barycenter introduced in

(5; 6). A k-barycenter of probabilities $\{\mu_1, \dots, \mu_n\}$ in $\mathcal{P}_2(\mathbb{R}^d)$ with weights $\lambda_1, \dots, \lambda_n$ is any k-set $\{\bar{\mu}_1, \dots, \bar{\mu}_k\}$ in $\mathcal{P}_2(\mathbb{R}^d)$ such that for any $\{\nu_i, \dots, \nu_k\} \subset \mathcal{P}_2(\mathbb{R}^d)$ we have that

$$\sum_{i=1}^{n} \lambda_i \min_{j \in \{1,\dots,k\}} \mathcal{W}_2^2(\mu_i, \bar{\mu}_j) \leq \sum_{i=1}^{n} \lambda_i \min_{j \in \{1,\dots,k\}} \mathcal{W}_2^2(\mu_i, \nu_j). \tag{3}$$

An α-trimmed k-barycenter of $\{\mu_1, \dots, \mu_n\}$ with weights as before is any k-set $\{\bar{\mu}_1, \dots, \bar{\mu}_k\}$ with weights $\bar{\lambda} = (\bar{\lambda}_1, \dots, \bar{\lambda}_n) \in \Lambda_\alpha(\lambda)$ such that

$$\sum_{i=1}^{n} \bar{\lambda}_i \min_{j \in \{1,\dots,k\}} \mathcal{W}_2^2(\mu_i, \bar{\mu}_j) = \min_{\{\nu_1,\dots,\nu_k\} \subset \mathcal{P}_2(\mathbb{R}^d), \lambda^* \in \Lambda_\alpha(\lambda)} \sum_{i=1}^{n} \lambda_i^* \min_{j \in \{1,\dots,k\}} \mathcal{W}_2^2(\mu_i, \nu_j), \tag{4}$$

where $\Lambda_\alpha(\lambda) = \{\lambda^* = (\lambda_1^*, \dots, \lambda_n^*) : 0 \leq \lambda_i^* \leq \lambda_i/(1-\alpha), \sum_{i=1}^n \lambda_i^* = 1\}$.

Broadly speaking k-barycenters can be thought of as an extension of k-means to the space of probabilities with finite second order, since we can rewrite (3) as

$$\min_{\mathfrak{S}} \sum_{j=1}^{k} \sum_{\mu_i \in \mathfrak{S}_j} \lambda_i \mathcal{W}_2^2(\mu_i, \bar{\mu}_j) \tag{5}$$

where $\mathfrak{S} = \{\mathfrak{S}_1, \dots, \mathfrak{S}_k\}$ is a partition of $\{\mu_1, \dots, \mu_n\}$ and $\bar{\mu}_j$ is the barycenter of the elements in $\mathfrak{S}_j$. Therefore, trimmed k-barycenters may be matched to trimmed k-means. As stated in (4), efficient computations can be done when dealing with location-scatter families of absolutely continuous distributions in $\mathcal{P}_2(\mathbb{R}^d)$. A notable example being the family of multivariate Gaussian distributions.

References

[1] Villani, C.: Optimal Transport: Old and New. Springer, (2009)

[2] Bertsimas, D., Tsitsiklis, J.: Introduction to Linear Optimization. Athena Scientific, (1997)

[3] Cuturi, M., Doucet, A.: Fast computation of wasserstein barycenters. PMLR 32, 685–693 (2014)

[4] del Barrio, E., Cuesta-Albertos, J., Matrán, C., Mayo-Íscar, A.: Robust clustering tools based on optimal transportation. Statistics and Computing **29**, 139–160 (2019)

[5] Boissard, E., Le Gouic, T., Loubes, J.-M., *et al.*: Distribution's template estimate with wasserstein metrics. Bernoulli **21**(2), 740–759 (2015)

[6] Gouic, T.L., Loubes, J.: Existence and consistency of wasserstein barycenters. Probab Theory Rel **168**, 901–917 (2017)
